# An fMRI-compatible force measurement system for the evaluation of the neural correlates of step initiation

**DOI:** 10.1038/srep43088

**Published:** 2017-02-23

**Authors:** Andrea Cristina de Lima-Pardini, Raymundo Machado de Azevedo Neto, Daniel Boari Coelho, Catarina Costa Boffino, Sukhwinder S. Shergill, Carolina de Oliveira Souza, Rachael Brant, Egberto Reis Barbosa, Ellison Fernando Cardoso, Luis Augusto Teixeira, Rajal G. Cohen, Fay Bahling Horak, Edson Amaro

**Affiliations:** 1 NIF - Neuroimagem Funcional, LIM44 - Laboratório de Investigação Médica 44 - LIM - 44, Department of Radiology, University of São Paulo, São Paulo, Brazil; 2 Human Motor Systems Laboratory, School of Physical Education and Sport, University of São Paulo, São Paulo, Brazil; 3 Department of Psychosis Studies, King’s College London, London, UK; 4 Movement Disorders Clinic, Department of Neurology, Hospital das Clínicas of the University of São Paulo School of Medicine, São Paulo, Brazil; 5 Department of Psychology & Communication Studies, University of Idaho, Moscow, Idaho, USA; 6 Department of Neurology, Oregon Health and Science University, Portland, Oregon, USA

## Abstract

Knowledge of brain correlates of postural control is limited by the technical difficulties in performing controlled experiments with currently available neuroimaging methods. Here we present a system that allows the measurement of anticipatory postural adjustment of human legs to be synchronized with the acquisition of functional magnetic resonance imaging data. The device is composed of Magnetic Resonance Imaging (MRI) compatible force sensors able to measure the level of force applied by both feet. We tested the device in a group of healthy young subjects and a group of elderly subjects with Parkinson’s disease using an event-related functional MRI (fMRI) experiment design. In both groups the postural behavior inside the magnetic resonance was correlated to the behavior during gait initiation outside the scanner. The system did not produce noticeable imaging artifacts in the data. Healthy young people showed brain activation patterns coherent with movement planning. Parkinson’s disease patients demonstrated an altered pattern of activation within the motor circuitry. We concluded that this force measurement system is able to index both normal and abnormal preparation for gait initiation within an fMRI experiment.

Any successful voluntary movement requires that we counteract the perturbations caused by the self-induced movement. This aspect of postural control, called *anticipatory postural adjustment (APA)*, is automatically triggered before the self-induced movement, and produces forces opposite to the biomechanical effects of the body perturbation. For instance, when we want to start walking, a combination of forces is required to move the center of mass forward. First, body weight is transferred to the support leg, followed by the withdrawal of the other heel from the floor. APAs have been thoroughly investigated in behavioral studies, and these have informed us about differences between normal and impaired gait initiation^1^,^2^. However, understanding how this seemingly effortless coordinated movement pattern is generated by the healthy and diseased nervous system has been a challenge to conventional neuroimaging. Most studies assessing brain function related to APAs have used manual tasks^3^,^4^, speech articulation^5^, and ankle dorsiflexion and plantiflexion^6^. Results of these studies consistently indicate a role for the supplementary motor area (SMA) in the preparation and organization of voluntary movements. It is possible that SMA also contributes to APAs during gait initiation^7^. However, the involvement of SMA and other brain structures during gait initiation in humans is still not clear. Investigating gait initiation is important, since it combines motor and cognitive components of movement preparation^8^. Indeed, difficulty with gait initiation is among the most incapacitating symptoms present in some neurological disorders, like Parkinson’s disease (PD)^7^,^8^,^9^.

Previous studies have investigated postural control using low spatial resolution techniques, such as electroencephalography^3^. Functional magnetic resonance imaging is able to offer neurophysiological information with good spatial and reasonable temporal resolution^10^. However, its use in the investigation of postural control is limited to the extent that subjects are required to lie still and supine in the scanner. For this reason, postural control and gait have largely been investigated using mental imagery - self-imagery of walking in a upright position^11^. Perhaps the major limitation of using motor imagery to investigate postural control is the difficulty of identifying specific gait phases, such as when producing APAs before a step initiation. Another important limitation is the possibility that motor imagery is itself impaired in certain populations, such as PD patients^12^.

A recent proposal by Lomond *et al*.^13^ may offer a solution to this technical limitation in the use of fMRI in the investigation of APAs. The subject in the scanner is asked to raise one leg, either from the hip (leg raise unsupported), or from the knee (leg raise supported) while lying in a supine position. Just as step initiation involves a postural weight shift to the support foot to stabilize the body prior to a step^1^, a straight leg raise requires an increase of vertical force under the foot of the supporting leg to stabilize the pelvis. Also, just as APAs in the standing position are suppressed when the body has external support^14^,^15^, APAs associated with leg raise are suppressed when the moving leg is supported by a pad under the knee. In this earlier study^13^, we did not align the behavior and data collection during imaging acquisition.

The force measurement system (FMS) we present here is suitable for postural assessment in the MRI environment. Here we show that the FMS measurements obtained in the scanner corresponded with well established measures of step initiation outside the scanner; the device is suitable to assess the neural correlates of normal and abnormal gait behavior, and head movement artifacts were within acceptable limits in all subjects.

## Results and Discussion

First, we tested whether the presence of the FMS in the scanner interfered with the quality of the brain images. We used a water-filled phantom and collected sequences both with and without the FMS. The results showed no interference of the FMS on brain images, with consistent spatial and temporal resolution quality across conditions. We also tested the amount of head motion by assessing the mean displacement of the combined medio-lateral, antero-posterior and superior-inferior planes. In the young group, the average of absolute head motion was 0.12 mm (standard error: SE = 0.01, range: 0.08–0.28) when subjects used the knee support and 0.14 mm (SE = 0.01, range: 0.07–0.26) unsupported (Wilcoxon Z = 24.5, p = 0.80). For the group with PD, mean amplitude of head motion when supported was 0.31 mm (SE = 0.02, range: 0.13–0.66) and 0.30 mm (SE = 0.01, range: 0.16–0.43) unsupported (Wilcoxon Z = 15, p = 0.72). The PD group showed greater amplitude of head movement (supported, Mann-Whitney U = 11, p = 0.01; unsupported, U = 9, p = 0.01). However, the level of head motion in both groups was within the conventional recommendation of absolute head movement less than 1 mm to avoid erroneous inference on neuronal activation in a 3 mm EPI voxel size^16^,^17^,^18^.

**Figure 1:**
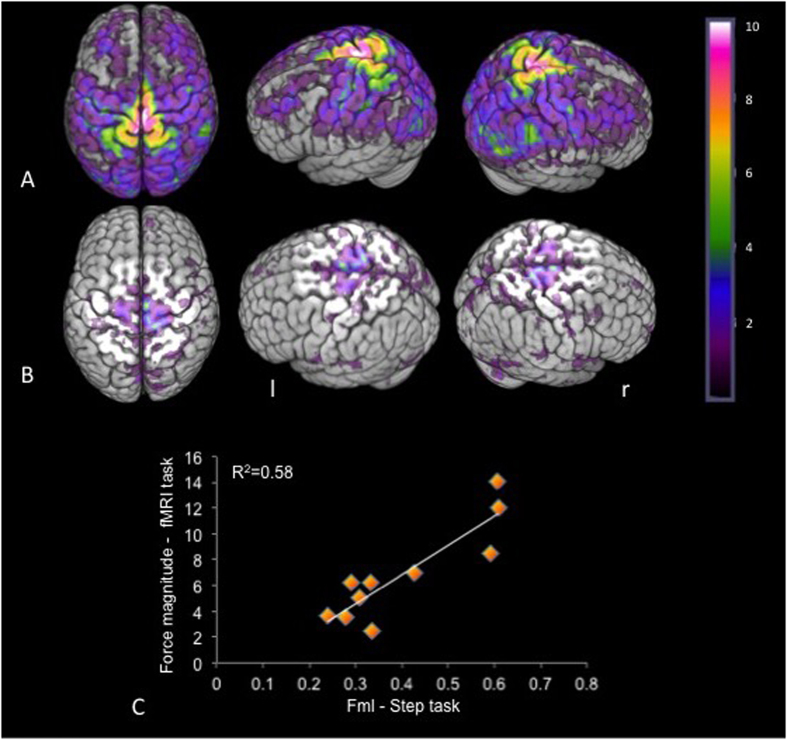
Frequency map (N = 10) of BOLD signal for the young subjects of the unsupported condition – the color bar indicates the number of overlapping BOLD signal (**A**). Frequency maps of the unsupported >supported contrast (**B**). Scatterplot of APA correlation performance on tasks performed inside and outside the scanner – difference of force applied by the support leg between the supported and unsupported conditions (**C**). (l = left; r = right).

**Figure 2:**
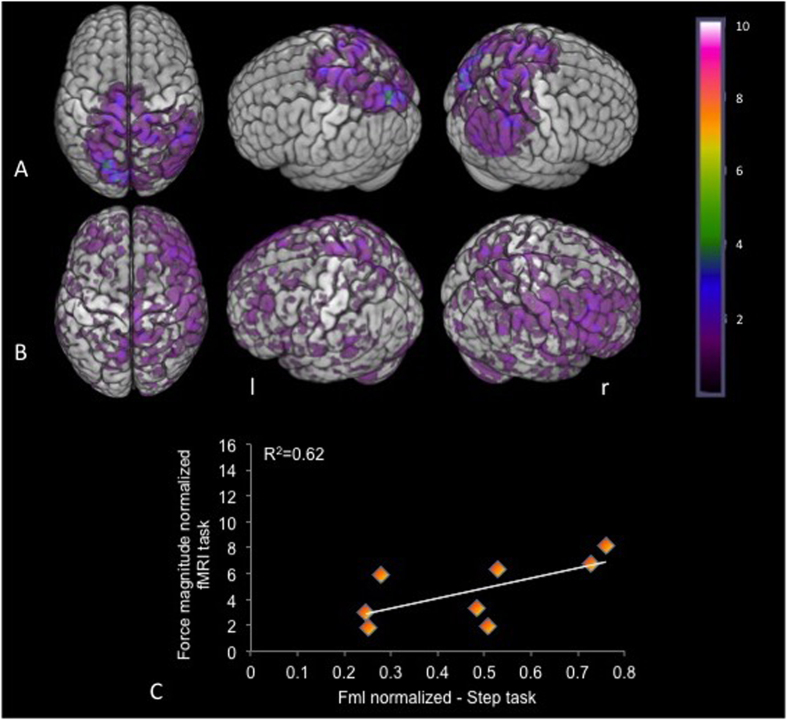
Frequency map (N = 8) of BOLD signal for the PD subjects of the unsupported condition – the color bar indicates the number of overlapping BOLD signal (**A**). Frequency maps of the unsupported >supported contrast (**B**). Scatterplot of APA correlation performance on tasks performed inside and outside the scanner – difference of force applied by the support leg between the conditions with and unsupported (**C**). (l = left; r = right).

We then assessed whether APAs produced in the scanner during supine leg raises (supported and unsupported) corresponded to APA production during upright gait initiation (step supported and unsupported). There were comparable levels of compensatory force applied by the support leg both inside and outside the scanner in healthy young and PD subjects, r_s_ (8) = 0.76 (p = 0.01); r_s_ (6) = 0.79 (p = 0.03), respectively (Figs 1C and 2C). The force applied by the left (support) leg during the movement (leg raise or step) was always lower in supported than in unsupported conditions, both inside and outside the scanner. The young group showed smaller amplitude of the medio-lateral force (F_ml_) in the supported (M = 0.38 N/cm, SE = 0.01, range = 0.20–0.60) compared to the unsupported condition (M = 0.80 N/cm, SE = 0.01, range = 0.70–0.88), Z = 0, p < 0.01). Also, the PD group had decreased amplitude of the F_ml_ in the supported (M = 0.41 N/cm, SE = 0.02, range = 0.11–0.74) compared to the unsupported condition (M = 0.88 N/cm, SE = 0.04, range = 0.36–1.50), Z = 0, p < 0.01). For the task in the scanner of the young group, the supported condition led to smaller values of the force magnitude (M = 4.20, SE = 0.13, range = 2.54–5.73) in comparison to the unsupported condition (M = 9.39, SE = 0.32, range = 6.14–14.10), Z = 0, P = 0.03. For the task in the scanner of the PD group, the supported condition led to smaller values of the force magnitude (M = 5.21, SE = 0.16, range = 3.02–7.33) in comparison to the unsupported condition (M = 7.80, SE = 0.21, range = 5.74–12.10), Z = 0, P = 0.03. Figure 1A shows the frequency maps of the young subjects during the unsupported condition (APA required). The maps evidence that most of the subjects had increased BOLD signal in medial regions of the pre and post central gyri and right SMA. This higher BOLD signal was also detected in the frequency map using the contrast (unsupported >supported) (Fig. 1B). The SMA is recognized to be involved in motor preparation^19^,^20^ for the performance of an intended movement. Therefore, not only the moving limb, but also synergies comprising both ipsilateral and contralateral body may be involved in the movement^2^,^21^. In fact, the bilateral SMA are reciprocally connected, projecting to both ipsilateral and contralateral primary motor cortex^22^,^23^. Both EEG and fMRI studies using connectivity analysis such as dynamic causal modeling (DCM) and effective connectivity networks (ECN) have shown bilateral interactions between SMA^24^,^25^,^26^ during unilateral manual tasks. These facts may explain the functional evidence of contralateral^24^,^27^,^28^ and bilateral^29^,^30^ SMA activation during simple unilateral hand and finger movements. Our protocol permits the isolation of the APA that occurs in the left leg from the intended movement of the right leg. Given that the left leg was mainly involved in the preparation of the APAs, the two potential possibilities were that there was either bilateral or right SMA activation. The bilateral sensorimotor and SMA cortices demonstrated an increased BOLD signal in both conditions (supported and unsupported). However, the right SMA and sensorimotor cortices showed increased activation in the contrast unsupported >supported, more specifically related to the APA executed by the left leg, supporting similar results when the task separated support from the direct limb movement^7^,^31^. An alternative interpretation for the increased activation of the right SMA during APA is that the movement assessed in the scanner required bilateral coordination, which has been shown to be associated with increased activation of the right SMA^32^,^33^.

**Figure 3:**
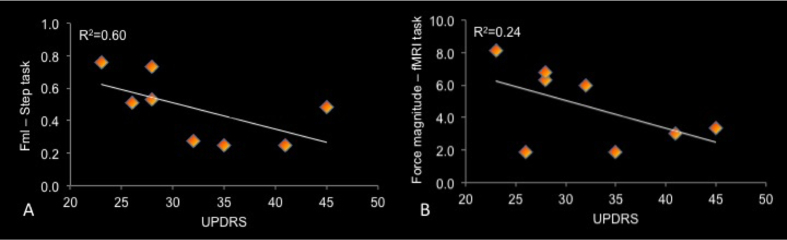
Scatterplot of the correlation between UPDRS and the APA (Fml) of the step task (**A**), and UPDRS and force magnitude of the leg lifting task (**B**) of the patients with PD. APA values are the difference between the unsupported and supported conditions.

The distribution of individual functional brain activation (frequency maps) for the unsupported condition (Fig. 2A) was sparse in the parietal-frontal areas in PD group. The frequency maps using the contrast (unsupported >supported) (Fig. 2B) did not show the involvement of any consistent regions. Group analysis for the contrast of interest (unsupported >supported) in the PD group did not demonstrate any regions reaching statistical significance. The lack of any focused pattern of brain activation in the PD group supports the premise that PD results in the involvement of an increased number of brain areas to accomplish a specific motor task, relative to healthy people^34^. Notably, there was a correlation of the UPDRS score of the PD group with the step initiation task r_s_ (6) = −0.78 (p = 0.02). The UPDRS correlation with the fMRI was similar to the correlation with the step task (scatterplots), however, the analysis did not reach statistical significance r_s_ (6) = −0.50 (p = 0.20) (Fig. 3). These results suggest that the severity of the disease impacts the amplitude of the APA as shown previously^1^,^35^. It is interesting that the performance of the task inside the scanner followed the behavior shown in the step initiation task, with the relationship of both variables (UPDRS and force magnitude), which supports the idea that the tasks share important behavioral components.

The FMS was shown to be valid for assessing postural control in healthy and PD populations in an fMRI environment allowing synchronized brain acquisition. The task performed in the scanner was highly correlated with gait initiation in different populations, showing brain BOLD signal coherent with known brain areas involved in motor control relevant to the circuitry associated with gait initiation^8^,^11^. Brain mechanisms associated with postural control have been previously studied only indirectly, which compromises the interpretation of the related neural circuitries^11^,^36^. Our method allow for the assessment of a special postural adjustment, composed of cognitive and movement components that could be explored in depth using several methods of brain function analysis (dynamic brain connectivity, machine learning)^37^,^38^, given that the time and spatial characteristics of the task performance can be synchronized with the acquisition of the neuroimaging data. Also, the FMS offers a potential instrument to explore pathological brain function of a postural component (APA) that has been associated with the pathophysiology of important gait disturbances, such as freezing of gait^39^. The management of the gait freezing is not well controlled with the current medications, neurosurgery, and physical therapy interventions; however, a more detailed knowledge of the dynamics of the postural circuitry in the brains of patients who freeze during gait offers an opportunity not only to better understand the mechanism, but also to consider more efficient treatments. The device and task developed by our team offers valid data on the neural correlates of postural control corresponding closely with the behavior of gait initiation.

The main limitation of the paradigm proposed here, common to all similar fMRI studies, is the lack of several features of postural control that are normally present in a natural environment – including gravitational influence^40^. Both step initiation and leg lifting require APAs of the support leg to release the self-initiated movement of the opposite leg^1^,^13^. In addition, the APA is decreased or suppressed in both tasks if the body is externally supported as showed in our results and previous studies^14^,^15^. We not only showed high correlation of the APA between the tasks, but also that this behavior is stable in two different populations. This data supports the hypothesis that the relative amplitude of APA triggered in advance of the opposite foot releasing is not influenced by the body position. In addition, our results in healthy young people showed increased activation of the SMA when APA was required, as observed in earlier studies of imagery of gait initiation in an fMRI protocol^41^ and in real gait initiation under EEG^42^ and TMS assessment^7^. Therefore, our functional brain results are in accordance with the existing data on neural correlates of gait initiation. Despite the inherent limitations of using fMRI to investigate postural control and gait, our fMRI task is the best model that we could offer to assess the coupling between APA and voluntary movements during movement initiation of the leg. Recent fMRI studies have also tried to overcome such limitations proposing tasks that resemble gait^43^ with new devices^44^,^45^ or imagery^11^,^46^. However, differently from earlier fMRI studies on postural control and gait, we demonstrated that the neural correlates of a task performed lying supine shares important postural aspects coupled to components with an ecological upright movement. Therefore, we believe that the behavioral relationship between these tasks could shed light on the neural correlates of gait initiation and its disorders.

We conclude that the force measurement system is an instrument compatible with fMRI and suitable to explore the neural correlates of both normal and abnormal gait initiation. The force measurement system (1) did not interfere with the quality of the fMRI; (2) could be used in event-related protocols in fMRI; (3) yielded data that were highly correlated with the gait initiation task. This system could become a useful tool for efficient design of postural control assessment in fMRI protocols, elucidating normal and pathological mechanisms of gait.

## Material and Methods

### Participants

**Figure 4:**
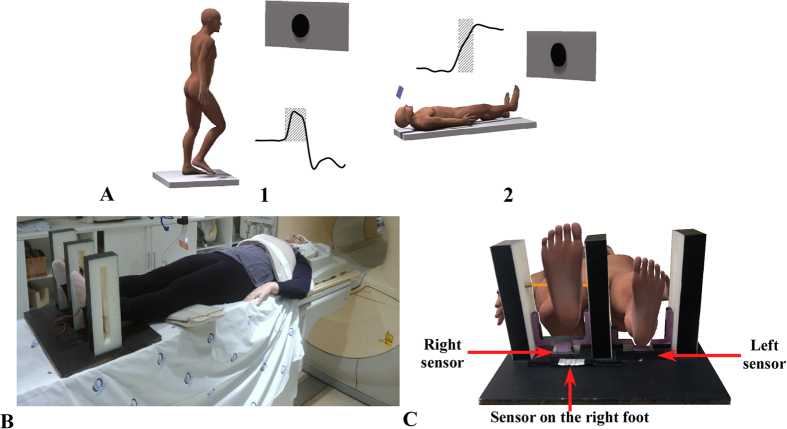
Compatible fMRI environment force measurement system (FMS). Example of the APA (highlighted area of each curve) measured during the step initiation (from a force platform) (**A1**) and during the leg raise task in the scanner (**A2**) using the FMS. Position of the FMS in the scanner (**B**). The FMS is composed by strain gage transducers (Flexiforce) embedded into a wooden base of support. There are one sensor under each foot and one sensor on the ankle of the right foot to record the moment that it touches the bar. Nylon bars separate and accommodate the feet, and a brass adjustable bar restricts the movement of the leg (**C**). The bands and Velcro straps to place the feet are omitted to make the figure clearer.

Ten right-handed healthy young subjects (mean age = 26.36 y, SD = 9.38 y; mean weight = 67.00 Kg, SD = 14.50 Kg; mean height = 1.75 m, SD = 0.10 m) without any muscular or neurological disorders participated in this study. We also tested the present protocol in eight people diagnosed with PD without freezing of gait as assessed by the New Freezing of Gait Questionnaire (NFoG-Q)^47^ and without any other diagnosed neurological or orthopedic/rheumatic impairments. The patients were classified as moderate by scoring 3 at Hoehn & Yahr^48^ scale, showing bilateral motor impairment and disturbance of body equilibrium. They were also assessed by the gold standard Unified Parkinson´s Disease Rating Scale – UPDRS)^49^ part III to classify their level of motor impairments considering bradykinesia, rigidity, tremor, balance and gait. All PD subjects were assessed ON medication (while taking their usual antiparkinsonian medication). The general profile and clinical information of each patient is depicted in Table 1. All participants provided informed consent, and experimental procedures were approved by the Institutional Review Board of the University of São Paulo. An informed consent to publish Fig. 4B was obtained. All experiments were performed in accordance with the Declaration of Helsinki^50^.

### Construction and implementation of the new compatible system to assess postural preparation in an MRI environment – Force Measurement System (FMS) for Postural Assessment in fMRI

Since it is not possible to measure postural preparation (APA) for step initiation during fMRI, we designed a task that simulates step initiation with and without external support in a supine position, that is feasible to be assessed during an fMRI protocol^13^. The task is to raise one leg, either from the hip with straight knee (condition that elicits APA) or from the supported knee (condition that suppress APA). The knee support was made by pads and small pillows to adjust the knee flexion to approximately 30 degrees. In order to investigate brain function in the exact moment of the APA during the protocol of leg lift, we designed an MRI-compatible force measurement system. The system comprises a structure custom-fit to the subject equipped with a pressure detector system made of a thin film polymer that is attached to a wooden base. A hardware box located outside the scanner room synchronizes data acquisition with stimulus presentation and stores data. Foot movement can be constrained by an adjustable height horizontal bar (Fig. 4B,C).

### Internal structure of the FMS

The program, written in C/C++ and executed in Linux LINT (customized version for microcomputer) operating system, acquires resistive signals from the sensors. The developmental interface is a Raspberry™ processor that reads and stores the signals and conveys them to Psychtoolbox-Matlab software in a MacBook Pro 13-inch, which presents instructions to the subjects. The processor has a memory card of 32 GB where the operating system and C/C++ code are stored, an output to the serial port, which conveys and receives the information from Matlab, a connection to an LCD alphanumeric monitor 20 × 4 cm that displays the forces measured in real time and a recorder, through a 4 GB USB.

Two hundred milliseconds before the display of the command to raise the leg, a signal is automatically sent (through a Matlab routine) to the measurement system to initiate the measurement of the forces of the sensors (under feet and above the movement leg). If the value is less than the mean minus 20 SD (decrease of force under the moving leg) the system sends a command to Matlab, and the command to the participant to relax is displayed after a time interval of 500 ms. Data from the three sensors are stored in a text file in the control box of the FMS. These data are recorded in the USB after the end of the BOLD sequence. The acquisition frequency of the sensors is 32 Hz.

### Position of the patient in the scanner using the FMS

In order to prevent head movements due to leg raise during the protocol, we physically constrained the patient’s head motion by placing a vacuum pillow around their head, pads behind the neck, and tapes on the chin and on the forehead. Also, bands with Velcro restricted motion of the upper trunk. Feet were positioned with one heel on each sensor of the wooden structure (Fig. 4B,C). The sensor under the right foot conveys information to the FMS on the moment that the foot is raised. A touch sensor is placed on the right foot, which sends a signal to the FMS when the foot touches an adjustable brass bar placed 1 cm above (restricting the amplitude of the foot movement, to reduce the amount of head movement provoked). Figure 4A2 depicts an example of the force output of the sensor under the left leg during the leg raise task unsupported. The highlighted area represents the APA.

### Biomechanical analysis of the step initiation

We quantified APAs during step initiation to associate the behavior in upright position with behavior in the supine task during the fMRI exam (Fig. 4A1). Participants were to take a step with the right foot. Both feet were positioned on a force platform. (AMTI OR6-6), with an internal analog low-pass filter with a cutoff frequency of 100 Hz. Subjects were instructed to take a step in two conditions: standing either unsupported or supported - holding a walker. Performing a step unsupported requires APAs^1^. On the other hand, holding an external support like a walker suppresses the APA^14^,^15^. A reflective marker was placed on the right malleolus to record the movement of the foot through a Vicon^®^ system (200 Hz, filter Butterworth low-pass 10 Hz). The participants performed 30 trials of step initiation in each condition. In each trial the participant was to follow the commands displayed on an LCD monitor (42″) positioned 3 m ahead: a cross, then an open or closed circle, followed by an open or closed circle. For example, participants were instructed to relax during the display of the cross, to prepare when they saw the open circle, and to step with the right foot when the closed circle was displayed. Half the participants observed the open circle as a ready cue and the other half observed the closed circle as a ready cue. Participants were instructed to stand with feet comfortably apart on the force plate, then the foot position was marked with tape. Instructions were to follow the commands on the monitor and to try to step as usual. After each trial the participant was to return to the initial position marked on the force plate. The order of conditions was counterbalanced across subjects.

### fMRI paradigm

Subjects performed one sequence of 30 trials in each condition (supported and unsupported) in an event-related paradigm. They observed three stimuli as presented in the biomechanical analysis outside the scanner: a cross – to relax, an open or closed circle – to prepare, and an open or closed circle – to raise the right leg (Fig. 4A2). The cross and the circles had two degrees in subtended visual angles. The order of the circles (open or closed) in the scanner followed what was performed during the biomechanical analysis of each patient. The duration of each stimulus was randomized and distributed according to the Poisson probability function to improve the detection of the hemodynamic response function (HRF) according to the Poisson distribution^51^,^52^: first circle (1 to 3 s, varying in steps of 0.5), second circle (up to 5 s – the stimulus disappears when the force under the movement leg drops), cross (interstimulus interval varying between 5.5 and 8.5 s).

### Image acquisition

Images acquisitions were made in a 3.0 T MR system (Achieva 3.0 T, Philips – The Netherlands) using a 32-channel head coil, (gradients of 80 mT/m). Visual stimuli were presented on a screen (2.5 m away) to a mirror above subjects’ eyes. Image acquisition was synchronized to data from FMS sensors using a light-coupled trigger system. BOLD images were acquired using T2*- weighted gradient echo EPI, SENSE acquisitions with the following parameters: TR = 2.000 ms, TE = 30 ms, 40 slices, 3.3 mm of slice thickness, 0.3 mm interslice gap, 3.3 mm isotropic voxels, 214 volumes (Total time: 6 m 58 s). Anatomical T1-weighted 3D images were used for reference and image registration (T1-FFE; TR = 7 ms, TE = 3.2 ms, 180 slices, FA = 8, 1 mm isotropic voxels).

### Image processing and behavior analysis

fMRI data was processed using FSL (www.fmrib.ox.ac.uk/fsl/)^53^. The volumes were processed by movement correction and calculation of mean displacement (MCFLIRT), spatial smoothing (FWHM = 5 mm) and spatial normalization to standard space (affine, 12 DoF)^54^,^55^. The activation maps were produced with the general linear model (GLM) using FILM routines based on semi-parametric estimation of residuals autocorrelation^56^. Group activation maps and group comparisons were obtained using a mixed-effects model, in order to include within-subject variances of parameter estimates. Significance was set at 1% for single-voxel level and at 5% (corrected) at mass-cluster level for group analyses.

### Behavioral analysis

The onset of the APA was defined as the time between the abrupt increase of the medio-lateral displacement - F_ml_ (2 standard deviations – SD above the mean of the baseline force) and the onset of the step, identified by the marker on the right malleolus (2 SD above the mean of the baseline foot displacement in the antero-posterior direction). The amplitude of the force during the step task was normalized by the size of the foot of the subjects (unit in N/cm). In the fMRI task, the magnitude of the force applied by the left foot on the sensor was calculated as the integer between the onset of the APA (2 SD above the mean of the baseline force) and the onset of the leg lifting (2 SD below the baseline force), normalized by the integer of the whole force curve, multiplied by 100. Nonparametric analyses were chosen due to the small sample size of both groups. Mean comparisons were performed using Wilcoxon for paired and Mann-Whitney U test for unpaired analysis and Spearman (r_s_) test for the correlations. The level of significance for behavioral parameters was set at 5%.

## Additional Information

**How to cite this article**: Lima-Pardini, A. C. *et al*. An fMRI-compatible force measurement system for the evaluation of the neural correlates of step initiation. *Sci. Rep.* **7**, 43088; doi: 10.1038/srep43088 (2017).

**Publisher's note:** Springer Nature remains neutral with regard to jurisdictional claims in published maps and institutional affiliations.

## Figures and Tables

**Table 1:** Patients demographics and clinical profile.

Subject	Age (y)	Gender	Weight (Kg)	Height (m)	Disease duration (years)	UPDRS (motor score)	DLeD (mg)	Side most affected
1	67	M	61	1.68	5	32	300	L
2	76	F	80	1.68	5	28	300	L
3	73	M	74	1.78	10	41	400	L
4	65	M	64	1.65	2.5	23	100	R
5	66	M	73	1.75	4	45	850	R
6	63	M	70	1.66	6	35	450	L
7	63	F	75	1.66	3	28	450	L
8	67	F	66	1.57	6	26	1100	L
Mean (±SD)	67.50 (4.66)	3F/5M	70.38 (6.35)	1.68 (6.40)	5.57 (2.22)	32.25 (7.63)	493.75 (324.52)	6L/2R

F: female, M: male. UPDRS: Unified Parkinson’s Disease Rating Scale. DLeD: Daily levodopa equivalent dose. L: left, R: right. M: mean, SD: standard deviation.

## References

[b1] Mancini M., Zampieri C., Carlson-Kuhta P., Chiari L. & Horak F. B. Anticipatory postural adjustments prior to step initiation are hypometric in untreated Parkinson’s disease: an accelerometer-based approach. Eur. J. Neurol. 16, 1028–1034 (2009). 19473350 10.1111/j.1468-1331.2009.02641.x PMC2840629

[b2] Aruin A. The organization of anticipatory postural adjustments. J. Autom. Control 12, 31–37 (2002).

[b3] Cunnington R., Windischberger C. & Moser E. Premovement activity of the pre-supplementary motor area and the readiness for action: studies of time-resolved event-related functional MRI. Hum. Mov. Sci. 24, 644–656 (2005). 16337295 10.1016/j.humov.2005.10.001

[b4] Ng T. H. B., Sowman P. F., Brock J. & Johnson B. W. Premovement brain activity in a bimanual load-lifting task. Exp. Brain Res. 208, 189–201 (2011). 21076820 10.1007/s00221-010-2470-5

[b5] Brendel B. . The contribution of mesiofrontal cortex to the preparation and execution of repetitive syllable productions: an fMRI study. NeuroImage 50, 1219–1230 (2010). 20080191 10.1016/j.neuroimage.2010.01.039

[b6] Francis S. . fMRI analysis of active, passive and electrically stimulated ankle dorsiflexion. NeuroImage 44, 469–479 (2009). 18950717 10.1016/j.neuroimage.2008.09.017

[b7] Jacobs J. V., Lou J. S., Kraakevik J. A. & Horak F. B. The supplementary motor area contributes to the timing of the anticipatory postural adjustment during step initiation in participants with and without Parkinson’s disease. Neuroscience 164, 877–885 (2009). 19665521 10.1016/j.neuroscience.2009.08.002 PMC2762010

[b8] Delval A., Tard C. & Defebvre L. Why we should study gait initiation in Parkinson’s disease. Neurophysiol. Clin. Neurophysiol. 44, 69–76 (2014). 10.1016/j.neucli.2013.10.127 24502907

[b9] Jacobs J. V., Nutt J. G., Carlson-Kuhta P., Stephens M. & Horak F. B. Knee trembling during freezing of gait represents multiple anticipatory postural adjustments. Exp. Neurol. 215, 334–341 (2009). 19061889 10.1016/j.expneurol.2008.10.019 PMC3141813

[b10] Amaro E. & Barker G. J. Study design in fMRI: basic principles. Brain Cogn. 60, 220–232 (2006). 16427175 10.1016/j.bandc.2005.11.009

[b11] Wai Y.-Y. . Cortical involvement in a gait-related imagery task: comparison between Parkinson’s disease and normal aging. Parkinsonism Relat. Disord. 18, 537–542 (2012). 22436654 10.1016/j.parkreldis.2012.02.004

[b12] Cohen R. G., Chao A., Nutt J. G. & Horak F. B. Freezing of gait is associated with a mismatch between motor imagery and motor execution in narrow doorways, not with failure to judge doorway passability. Neuropsychologia 49, 3981–3988 (2011). 22027173 10.1016/j.neuropsychologia.2011.10.014 PMC3260879

[b13] Lomond K. V. . Protocol to assess the neurophysiology associated with multi-segmental postural coordination. Physiol. Meas. 34, N97–N105 (2013). 24065623 10.1088/0967-3334/34/10/N97 PMC3884551

[b14] Schieppati M. & Nardone A. Free and supported stance in Parkinson’s disease: the effect of posture and “postural set”on leg muscle responses to perturbation, and its relation to the severity of the disease. Brain 114, 1227–1244. doi: 10.1093/brain/114.3.1227 (1991). 2065247

[b15] Chong R. K., Horak F. B. & Woollacott M. H. Parkinson’s disease impairs the ability to change set quickly. J. Neurol. Sci. 175, 57–70 (2000). 10785258 10.1016/s0022-510x(00)00277-x

[b16] Seto E. . Quantifying Head Motion Associated with Motor Tasks Used in fMRI. NeuroImage 14, 284–297 (2001). 11467903 10.1006/nimg.2001.0829

[b17] Glover G. H. & Lai S. Self-navigated spiral fMRI: interleaved versus single-shot. Magn. Reson. Med. 39, 361–368 (1998). 9498591 10.1002/mrm.1910390305

[b18] Field A. S., Yen Y. F., Burdette J. H. & Elster A. D. False cerebral activation on BOLD functional MR images: study of low-amplitude motion weakly correlated to stimulus. AJNR Am. J. Neuroradiol. 21, 1388–1396 (2000). 11003269 PMC7974040

[b19] Tanji J. New concepts of the supplementary motor area. Curr. Opin. Neurobiol. 6, 782–787 (1996). 9000016 10.1016/s0959-4388(96)80028-6

[b20] Hyland B., Chen D. F., Maier V., Palmeri A. & Wiesendanger M. What is the role of the supplementary motor area in movement initiation? Prog. Brain Res. 80, 431–436; discussion 427–430 (1989). 2699376 10.1016/s0079-6123(08)62240-2

[b21] Baldissera F. G. & Esposti R. The role of anticipatory postural adjustments in interlimb coordination of coupled arm movements in the parasagittal plane: II. Postural activities and coupling coordination during cyclic flexion-extension arm movements, ISO- and. Exp. Brain Res. 229, 203–219 (2013). 23793445 10.1007/s00221-013-3605-2

[b22] Bozkurt B. . Microsurgical and Tractographic Anatomy of the Supplementary Motor Area Complex in Humans. World Neurosurg. 95, 99–107 (2016). 27476690 10.1016/j.wneu.2016.07.072

[b23] Rouiller E. M. . Transcallosal connections of the distal forelimb representations of the primary and supplementary motor cortical areas in macaque monkeys. Exp. Brain Res. 102, 227–243 (1994). 7705502 10.1007/BF00227511

[b24] Gao Q., Tao Z., Zhang M. & Chen H. Differential Contribution of Bilateral Supplementary Motor Area to the Effective Connectivity Networks Induced by Task Conditions Using Dynamic Causal Modeling. Brain Connect. 4, 256–264 (2014). 24606178 10.1089/brain.2013.0194 PMC4028092

[b25] Deecke L. Bereitschaftspotential as an indicator of movement preparation in supplementary motor area and motor cortex. Ciba Found. Symp. 132, 231–250 (1987). 3322717 10.1002/9780470513545.ch14

[b26] Grefkes C., Eickhoff S. B., Nowak D. A., Dafotakis M. & Fink G. R. Dynamic intra- and interhemispheric interactions during unilateral and bilateral hand movements assessed with fMRI and DCM. NeuroImage 41, 1382–1394 (2008). 18486490 10.1016/j.neuroimage.2008.03.048

[b27] Fried I. . Functional organization of human supplementary motor cortex studied by electrical stimulation. J. Neurosci. Off. J. Soc. Neurosci. 11, 3656–3666 (1991). 10.1523/JNEUROSCI.11-11-03656.1991 PMC6575551 1941101

[b28] Yokoyama O., Nakayama Y. & Hoshi E. Area- and band-specific representations of hand movements by local field potentials in caudal cingulate motor area and supplementary motor area of monkeys. J. Neurophysiol. 115, 1556–1576 (2016). 26792884 10.1152/jn.00882.2015 PMC4808131

[b29] Lee K. M., Chang K. H. & Roh J. K. Subregions within the supplementary motor area activated at different stages of movement preparation and execution. NeuroImage 9, 117–123 (1999). 9918733 10.1006/nimg.1998.0393

[b30] Carlsen A. N., Eagles J. S. & MacKinnon C. D. Transcranial direct current stimulation over the supplementary motor area modulates the preparatory activation level in the human motor system. Behav. Brain Res. 279, 68–75 (2015). 25446764 10.1016/j.bbr.2014.11.009 PMC4857713

[b31] Viallet F., Massion J., Massarino R. & Khalil R. Performance of a bimanual load-lifting task by parkinsonian patients. J. Neurol. Neurosurg. Psychiatry 50, 1274–1283 (1987). 3681306 10.1136/jnnp.50.10.1274 PMC1032450

[b32] Sadato N., Yonekura Y., Waki A., Yamada H. & Ishii Y. Role of the supplementary motor area and the right premotor cortex in the coordination of bimanual finger movements. J. Neurosci. Off. J. Soc. Neurosci. 17, 9667–9674 (1997). 10.1523/JNEUROSCI.17-24-09667.1997 PMC6573404 9391021

[b33] Chan J. L. & Ross E. D. Left-handed mirror writing following right anterior cerebral artery infarction: evidence for nonmirror transformation of motor programs by right supplementary motor area. Neurology 38, 59–63 (1988). 3336465 10.1212/wnl.38.1.59

[b34] Caproni S. . Complexity of motor sequences and cortical reorganization in Parkinson’s disease: a functional MRI study. PloS One 8, e66834 (2013). 23825570 10.1371/journal.pone.0066834 PMC3692521

[b35] Aruin A. S., Neyman I., Nicholas J. J. & Latash M. L. Are there deficits in anticipatory postural adjustments in Parkinson’s disease? Neuroreport 7, 1794–1796 (1996). 8905667 10.1097/00001756-199607290-00021

[b36] Fasano A., Herman T., Tessitore A., Strafella A. P. & Bohnen N. I. Neuroimaging of Freezing of Gait. J. Park. Dis. 5, 241–254 (2015). 10.3233/JPD-150536 PMC4923721 25757831

[b37] Hutchison R. M. . Dynamic functional connectivity: Promise, issues, and interpretations. NeuroImage 80, 360–378 (2013). 23707587 10.1016/j.neuroimage.2013.05.079 PMC3807588

[b38] Sato J. R. . An fMRI normative database for connectivity networks using one-class support vector machines. Hum. Brain Mapp. 30, 1068–1076 (2009). 18412113 10.1002/hbm.20569 PMC6870648

[b39] Nutt J. G. . Freezing of gait: moving forward on a mysterious clinical phenomenon. Lancet Neurol. 10, 734–744 (2011). 21777828 10.1016/S1474-4422(11)70143-0 PMC7293393

[b40] Spironelli C., Busenello J. & Angrilli A. Supine posture inhibits cortical activity: Evidence from Delta and Alpha EEG bands. Neuropsychologia 89, 125–131 (2016). 27312745 10.1016/j.neuropsychologia.2016.06.015

[b41] Wang J. . Functional MRI in the assessment of cortical activation during gait-related imaginary tasks. J. Neural Transm. 116, 1087–1092 (2009). 19669694 10.1007/s00702-009-0269-y

[b42] Yazawa S. . Cortical mechanism underlying externally cued gait initiation studied by contingent negative variation. Electroencephalogr. Clin. Neurophysiol. 105, 390–399 (1997). 9363005 10.1016/s0924-980x(97)00034-9

[b43] Dobkin B. H., Firestine A., West M., Saremi K. & Woods R. Ankle dorsiflexion as an fMRI paradigm to assay motor control for walking during rehabilitation. NeuroImage 23, 370–381 (2004). 15325385 10.1016/j.neuroimage.2004.06.008 PMC4164211

[b44] Hollnagel C. . Brain activity during stepping: A novel MRI-compatible device. J. Neurosci. Methods 201, 124–130 (2011). 21827788 10.1016/j.jneumeth.2011.07.022

[b45] Snijders A. H. . Gait-related cerebral alterations in patients with Parkinson’s disease with freezing of gait. Brain 134, 59–72 (2011). 21126990 10.1093/brain/awq324

[b46] Peterson D. S., Pickett K. A., Duncan R., Perlmutter J. & Earhart G. M. Gait-related brain activity in people with Parkinson disease with freezing of gait. PloS One 9, e90634 (2014). 24595265 10.1371/journal.pone.0090634 PMC3940915

[b47] Nieuwboer A. . Reliability of the new freezing of gait questionnaire: Agreement between patients with Parkinson’s disease and their carers. Gait Posture 30, 459–463 (2009). 19660949 10.1016/j.gaitpost.2009.07.108

[b48] Hoehn M. M. & Yahr M. D. Parkinsonism: onset, progression and mortality. Neurology 17, 427–442 (1967). 6067254 10.1212/wnl.17.5.427

[b49] Fahn S., Marsden M., Goldstein M. & Calne D. *Recent Developments in Parkinson’s Disease*. **2** (1986).

[b50] World Medical Association. World Medical Association Declaration of Helsinki. Ethical principles for medical research involving human subjects. Bull. World Health Organ. 79, 373–374 (2001). 11357217 PMC2566407

[b51] Hagberg G. E., Zito G., Patria F. & Sanes J. N. Improved Detection of Event-Related Functional MRI Signals Using Probability Functions. NeuroImage 14, 1193–1205 (2001). 11697951 10.1006/nimg.2001.0880

[b52] Wager T. D. & Nichols T. E. Optimization of experimental design in fMRI: a general framework using a genetic algorithm. NeuroImage 18, 293–309 (2003). 12595184 10.1016/s1053-8119(02)00046-0

[b53] Smith S. M. . Advances in functional and structural MR image analysis and implementation as FSL. NeuroImage 23, S208–S219 (2004). 15501092 10.1016/j.neuroimage.2004.07.051

[b54] Jenkinson M., Bannister P., Brady M. & Smith S. Improved optimization for the robust and accurate linear registration and motion correction of brain images. NeuroImage 17, 825–841 (2002). 12377157 10.1016/s1053-8119(02)91132-8

[b55] Smith S. M. Fast robust automated brain extraction. Hum. Brain Mapp. 17, 143–155 (2002). 12391568 10.1002/hbm.10062 PMC6871816

[b56] Woolrich M. W., Ripley B. D., Brady M. & Smith S. M. Temporal autocorrelation in univariate linear modeling of FMRI data. NeuroImage 14, 1370–1386 (2001). 11707093 10.1006/nimg.2001.0931

